# Blood-nulled late gadolinium enhancement increases sensitivity for subtle basal myocardial scar in mitral valve prolapse with annular disjunction

**DOI:** 10.1016/j.radcr.2026.05.076

**Published:** 2026-06-22

**Authors:** Amy Avakian, Muhammad Umair

**Affiliations:** a Department of Radiology, Southern Hills Hospital and Medical Center, Las Vegas, NV, USA; b Department of Radiology, Columbia University, New York, NY, USA; c Russell H. Morgan Department of Radiology and Radiological Sciences, Johns Hopkins University, Baltimore, MD, USA

**Keywords:** Mitral valve prolapse, Mitral annular disjunction, Blood-nulled late gadolinium enhancement, Dark-blood LGE, Cardiac MRI, Myocardial fibrosis

## Abstract

Mitral valve prolapse (MVP) with mitral annular disjunction (MAD) is an increasingly recognized arrhythmogenic phenotype in which focal myocardial fibrosis of the basal inferolateral wall serves as a substrate for ventricular arrhythmia and a target for cardiac magnetic resonance (CMR) risk stratification, yet this fibrosis is often subtle and may be obscured on conventional bright-blood late gadolinium enhancement (LGE) imaging where subendocardial scar blends with the adjacent bright blood pool. We describe a 67-year-old woman with known MVP and a 3-mm posterior MAD referred for CMR following 24-hour Holter monitoring that documented frequent premature ventricular contractions, short runs of supraventricular tachycardia, and possible atrial tachycardia with intermittent variable atrioventricular block. Conventional phase-sensitive inversion recovery (PSIR) LGE was interpreted as negative on prospective review, with preserved global left ventricular systolic function and no regional wall motion abnormality. A matched dark-blood PSIR LGE sequence performed at identical slice prescription during the same examination demonstrated a focal mid-to-subendocardial stripe of enhancement in the basal inferolateral wall; with this finding in hand, a faint band of enhancement could be retrospectively identified at the same anatomical location on the conventional images, but had not met the threshold for confident prospective identification. The spatial distribution, in continuity with the disjunctive posterior annulus rather than conforming to a single coronary territory, favored an MVP-MAD-related substrate over an ischemic etiology, and the finding reinforced the indication for continued arrhythmic surveillance and supported electrophysiology consultation. To our knowledge, this is among the first published descriptions of incremental diagnostic yield from dark-blood PSIR LGE in MVP-MAD and supports inclusion of a dark-blood sequence in dedicated MVP-MAD CMR protocols.

## Introduction

Mitral valve prolapse (MVP) with mitral annular disjunction (MAD) is increasingly recognized as an arrhythmogenic entity associated with ventricular arrhythmia and rare but devastating sudden cardiac death [[1], [2], [3]]. Myocardial fibrosis, most commonly involving the basal inferolateral left ventricular wall and the posteromedial papillary muscle, is presumed to reflect chronic mechanical stress at the disjunctive annulus and to serve as the structural substrate for electrical instability in this population [1,2,4].

Cardiac magnetic resonance (CMR) imaging enables noninvasive detection of myocardial fibrosis through late gadolinium enhancement (LGE), and the presence of scar has been associated with ventricular arrhythmia and adverse outcomes in MVP [[3], [4], [5]]. However, conventional inversion-recovery LGE may have limited sensitivity for subtle or non-transmural fibrosis in basal segments, where thin subendocardial scar can blend with the adjacent bright blood pool. Blood-nulled (dark-blood) LGE techniques suppress left ventricular cavity signal while preserving scar enhancement and have been shown to improve sensitivity for subendocardial fibrosis in ischemic and selected non-ischemic substrates [[6], [7], [8], [9], [10]]. Their incremental value in the specific context of MVP-MAD has, to our knowledge, received limited dedicated description. We present a case in which prospective conventional PSIR LGE imaging was interpreted as negative, but a matched dark-blood PSIR LGE sequence demonstrated a focal mid-to-subendocardial stripe of enhancement in the basal inferolateral wall in a patient with MVP and posterior MAD.

## Clinical presentation

A 67-year-old woman with echocardiographically confirmed mitral valve prolapse and a 3-mm posterior mitral annular disjunction was referred for cardiac MRI for evaluation of palpitations and documented arrhythmia. Twenty-four-hour ambulatory Holter monitoring demonstrated frequent premature ventricular contractions and several short runs of supraventricular tachycardia, with episodes felt to be most consistent with atrial tachycardia and intermittent variable atrioventricular block. No sustained ventricular tachycardia or non-sustained ventricular tachycardia runs were captured during the monitoring period. The patient denied chest pain and anginal-equivalent symptoms. Prior transthoracic echocardiography characterized the MVP as single-leaflet posterior prolapse with leaflet thickening; no significant mitral regurgitation was documented on the index echocardiogram. Given the documented arrhythmia burden and the established association between MVP-MAD and arrhythmic events, CMR was performed to evaluate for underlying myocardial fibrosis as an arrhythmogenic substrate.

## Imaging findings

### Cardiac MRI protocol

Cardiac MRI was performed on a 1.5 T clinical scanner. The protocol included cine balanced steady-state free precession (bSSFP) imaging in standard short-axis, 2-, 3-, and 4-chamber long-axis planes for morphologic and functional assessment. Gadolinium contrast was administered intravenously as gadobutrol at 0.1 mmol/kg. Late gadolinium enhancement imaging was acquired beginning approximately 10 minutes after contrast administration. Conventional bright-blood LGE was performed using a segmented phase-sensitive inversion recovery (PSIR) sequence with breath-held single-shot bSSFP readout in short-axis stack and matching long-axis planes (slice thickness 8 mm, in-plane resolution approximately 1.4 × 1.4 mm, inversion time individualized using a TI scout). Dark-blood LGE was acquired immediately following the conventional acquisition at identical slice prescription, using a PSIR sequence modified with a T2-magnetization preparation pulse that exploits the shorter T2 of blood relative to enhanced myocardium to suppress LV cavity signal, as described by Kellman et al. [5]. Identical inversion time, slice thickness, and matrix were used to permit direct visual comparison with the conventional images. Native T1 mapping and post-contrast extracellular volume calculation were not performed in this examination.

### Morphologic and functional assessment

Cardiac MRI confirmed mitral valve prolapse with posterior leaflet thickening and a 3-mm posterior mitral annular disjunction (Fig. 1). The MAD was measured on the end-systolic frame of the 3-chamber long-axis cine, perpendicular to the long axis of the left ventricle, from the hinge point of the posterior mitral leaflet at the atrial wall to the crest of the basal inferolateral myocardium, in accordance with the convention described by Faletra et al. [8]. Global left ventricular systolic function was preserved and no regional wall motion abnormality was identified.

**Fig. 1:**
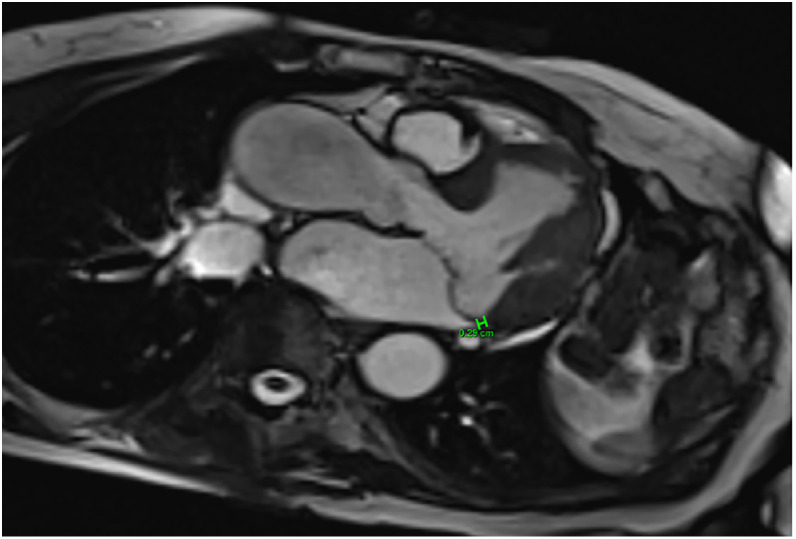
Mitral valve prolapse with posterior annular disjunction on cine imaging. Three-chamber long-axis cine cardiac MRI, end-systolic frame, demonstrating mitral valve prolapse with posterior mitral annular disjunction measuring approximately 3 mm (caliper). The measurement was performed perpendicular to the long axis of the left ventricle, from the hinge point of the posterior mitral leaflet at the atrial wall to the crest of the basal inferolateral myocardium. Abbreviation: MAD, mitral annular disjunction.

### Conventional late gadolinium enhancement

Conventional bright-blood PSIR LGE was interpreted as negative on prospective clinical review, with no visually apparent myocardial enhancement on the basal short-axis or 4-chamber long-axis acquisitions (Fig. 2A,C).

**Fig. 2:**
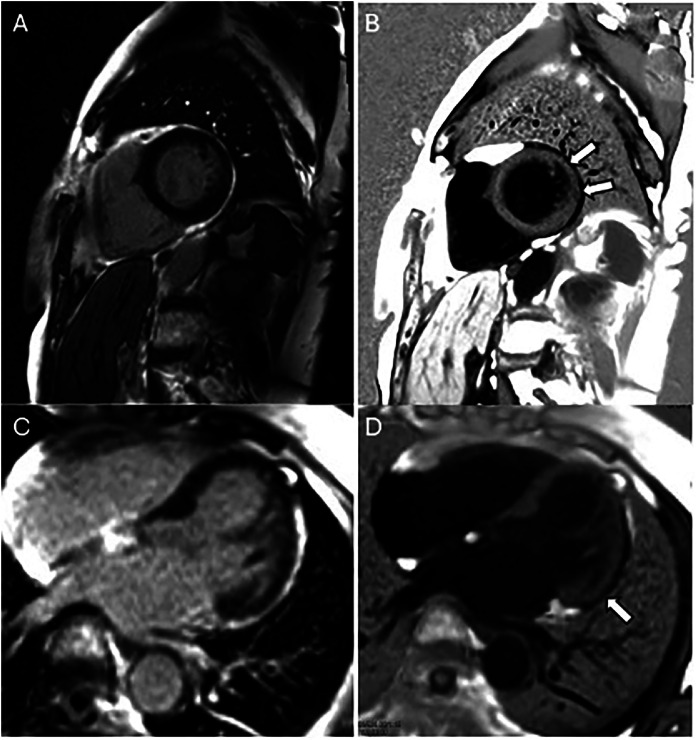
Conventional bright-blood late gadolinium enhancement (LGE) compared with matched blood-nulled (dark-blood) LGE. All panels acquired at identical slice prescription, cardiac phase, and grayscale window. (**A**) Conventional bright-blood PSIR LGE, basal short-axis. Prospective interpretation was negative for myocardial enhancement. (**B**) Matched dark-blood PSIR LGE, basal short-axis. Arrows indicate a focal mid-to-subendocardial stripe of enhancement in the basal inferolateral wall. (**C**) Conventional bright-blood PSIR LGE, 4-chamber long-axis. Prospectively interpreted as without myocardial enhancement. (**D**) Matched dark-blood PSIR LGE, 4-chamber long-axis. Arrow indicates the same anatomical focus of basal inferolateral mid-to-subendocardial enhancement. With knowledge of the dark-blood finding, a faint band of enhancement at the same location could be retrospectively appreciated on panels A and C, but had not met the threshold for confident prospective identification.

### Blood-nulled late gadolinium enhancement

On the matched dark-blood PSIR LGE images acquired at identical slice prescription, a focal mid-to-subendocardial stripe of enhancement was identified along the basal inferolateral left ventricular wall, seen on both the basal short-axis (Fig. 2B) and 4-chamber long-axis (Fig. 2D) reconstructions. With knowledge of this finding, a faint and ill-defined band of enhancement at the same anatomical location could be appreciated on retrospective review of the conventional PSIR images, but had not met the threshold for confident prospective identification because of marginal contrast against the adjacent blood pool. No additional enhancement was identified elsewhere in the left ventricle or in the papillary muscles.

### Ischemic differential

Although mid-to-subendocardial enhancement at the basal inferolateral wall can also reflect chronic ischemic injury within a left circumflex territory, several features favored a non-ischemic MVP-MAD-related substrate in this case. The patient lacked anginal and anginal-equivalent symptoms. The enhancement was confined to a small focus in direct continuity with the disjunctive posterior annulus rather than extending along a vascular territory, and this distribution matches the pattern repeatedly described in published MVP-MAD CMR series [1,2,4]. A formal exclusion of obstructive coronary disease by coronary CT angiography or invasive coronary angiography was not pursued, but the clinical and imaging features did not support an ischemic etiology.

### Management and follow-up

The CMR findings were discussed with the referring clinical team. The identification of focal basal inferolateral fibrosis on dark-blood LGE reinforced the indication for continued ambulatory rhythm surveillance and supported referral for electrophysiology consultation, given the documented arrhythmia burden and the published association between basal inferolateral fibrosis and arrhythmic events in MVP-MAD [1,3,6,7].

## Discussion

This case illustrates an incremental diagnostic yield of dark-blood phase-sensitive inversion recovery LGE over conventional bright-blood PSIR LGE for the detection of subtle basal subendocardial myocardial fibrosis in MVP with mitral annular disjunction. To our knowledge, this is among the first published descriptions of this incremental yield in the specific context of MVP-MAD risk stratification.

### Mechanisms of false-negative conventional LGE in the basal segments

Conventional segmented inversion-recovery and PSIR LGE acquisitions are optimized to null normal remote myocardium, leaving the left ventricular cavity bright. As a consequence, scar-to-myocardium contrast is preserved but scar-to-blood contrast is compromised. Thin subendocardial fibrosis can become isointense with, or partially obscured by, the adjacent bright blood pool, particularly when the lesion is mid-to-subendocardial in extent, when the inversion time is even slightly mistuned for the time after contrast administration, or when through-plane partial-volume averaging at curved basal segments blurs the blood–myocardium border [5,9]. Slow-flow artifact along the inferolateral wall and the oblique through-plane orientation of the basal short-axis stack further reduce conspicuity in the precise region of interest in MVP-MAD.

### Dark-blood PSIR LGE: technical basis and advantages

Several dark-blood LGE strategies have been described [5,9,10]. The implementation used in this case prepends a T2-magnetization preparation pulse to a PSIR acquisition and exploits the shorter T2 of blood relative to enhanced myocardium. This suppresses the LV cavity signal while preserving the magnetization of enhanced myocardium, producing scar that appears bright against both dark remote myocardium and dark blood pool. The dual-contrast effect underlies the reported gains in subendocardial scar conspicuity in ischemic substrates [5,9] and in selected non-ischemic substrates [10]. The present case extends this practical advantage to subtle MVP-MAD-related fibrosis. Importantly, the additional sequence adds only a small increment to total scan time at identical slice prescription, which facilitates direct visual comparison and integration into existing CMR protocols.

### Clinical significance

The arrhythmogenic potential of MVP with annular disjunction is well established [[1], [2], [3],^6^]. Pathologic and clinical studies have demonstrated focal myocardial scarring at the basal inferolateral wall and the posteromedial papillary muscle as a key substrate for electrical instability, attributed to chronic mechanical traction on the disjunctive annulus and on the papillary muscle apparatus [1,2,4]. LGE-detected fibrosis in this distribution has been associated with ventricular arrhythmia and adverse outcomes, and is incorporated into current expert consensus recommendations for the evaluation of arrhythmic MVP.⁷ In the present case, the dark-blood LGE finding reinforced clinical risk surveillance and supported electrophysiology referral that would have been less strongly indicated based on the prospectively negative conventional LGE alone. This sequence of events supports inclusion of a dedicated dark-blood LGE sequence in MVP-MAD CMR protocols when arrhythmic risk stratification is the indication.

### Limitations

This is a single-case observation and cannot establish the diagnostic accuracy of dark-blood PSIR LGE in MVP-MAD as a population. The reference standard for myocardial fibrosis is histopathology, which was not available in this in vivo setting; the dark-blood finding cannot therefore be independently confirmed. The retrospective re-review of the conventional LGE images was not blinded, which biases identification toward concordance. Quantitative comparison of scar-to-blood contrast-to-noise ratio between the 2 sequences was not performed in this case but would be a logical next step in prospective cohort study. Native T1 mapping and post-contrast extracellular volume calculation, which can complement focal LGE assessment by characterizing diffuse fibrosis, were not performed.

## Conclusion

In a 67-year-old woman with mitral valve prolapse and posterior mitral annular disjunction referred for evaluation of palpitations and documented arrhythmia, dark-blood PSIR late gadolinium enhancement demonstrated a focal mid-to-subendocardial stripe of enhancement in the basal inferolateral wall that was not confidently identified on prospective conventional PSIR LGE. To our knowledge, this is among the first descriptions of incremental diagnostic yield from dark-blood PSIR LGE in MVP-MAD. Inclusion of a dedicated dark-blood LGE sequence in MVP-MAD CMR protocols may improve sensitivity for the subtle basal fibrosis that anchors arrhythmic risk stratification in this population, at the cost of only a small increment in total scan time.

## Author contributions

AA contributed to literature review, drafting of the manuscript, and preparation of figures and figure legends. UM contributed to interpretation of imaging findings, advanced cardiac MRI protocol guidance, imaging interpretation, critical revision of the manuscript for important intellectual content, and overall supervision of the project. Both authors reviewed and approved the final manuscript and agree to be accountable for all aspects of the work.

## Patient consent

Written informed consent was obtained from the patient for publication of her clinical details and cardiac MRI images in this case report. The patient was informed that the case would be published in a medical journal for educational purposes. The signed consent documentation is retained by the authors.
